# Supplementary material for: The Stress-Dependent Dynamics of Saccharomyces cerevisiae tRNA and rRNA Modification Profiles
Source: Genes (Basel). 2021 Aug 28;12(9):1344. doi: 10.3390/genes12091344 (PMC8470187; doi:10.3390/genes12091344)
Supplement: Supplementary file 1 [file genes-12-01344-s001.zip › genes-1301375-supplementary.pdf]

genes MDPI

Article

Stress-Dependent Dynamics of the *S. cerevisiae* tRNA and rRNA Modification Profile

Yasemin Yoluç [1], Erik van de Logt [1,2] and Stefanie Kellner [1,*]

[1] Department of Pharmaceutical Chemistry, Goethe University Frankfurt, Max-von-Laue str 9, 60438 Frankfurt; yoluc@pharmchem.uni-frankfurt.de
[2] Department of Chemistry, Ludwig-Maximilians University Munich, Butenandtstr. 5-13, 81377 Munich, Germany; vandelogt@genzentrum.lmu.de
* Correspondence: Kellner@pharmchem.uni-frankfurt.de; Tel.: +49-069-798-29919

Content:

Figure S1: LD_{50} for S. cerevisiae BY4741
Figure S2: detected damage products in total RNA after MMS treatment
Figure S3: comparative NAIL-MS for labeling validation of $^{13}C_6$-glucose and $^{15}N_2$-uracil labeling
Figure S4: comparison of $SILIS^{Gen1}$ and $SILIS^{Gen2}$
Figure S5: High-resolution mass spectra of canonical nucleosides in $SILIS^{Gen2}$
Figure S6: Methylome discrimination based pulse chase experiment, modification levels of m^3C and m^7G
Figure S7: examination of knock out strains
Figure S8: Absolute abundance of modified nucleosides per original tRNA from NAIL-MS pulse chase experiment.
Figure S9: NAIL-MS pulse chase experiment, levels of i6A and t6A after AsO_2 and TBH treatment

Table S1: Overview of used synthetic standards
Table S2: absolute number of enzymatically and damage methylated nucleosides in 25S, 18S rRNA and tRNA
Table S3: absolute modifications levels in total RNA after MMS treatment in knock out strains

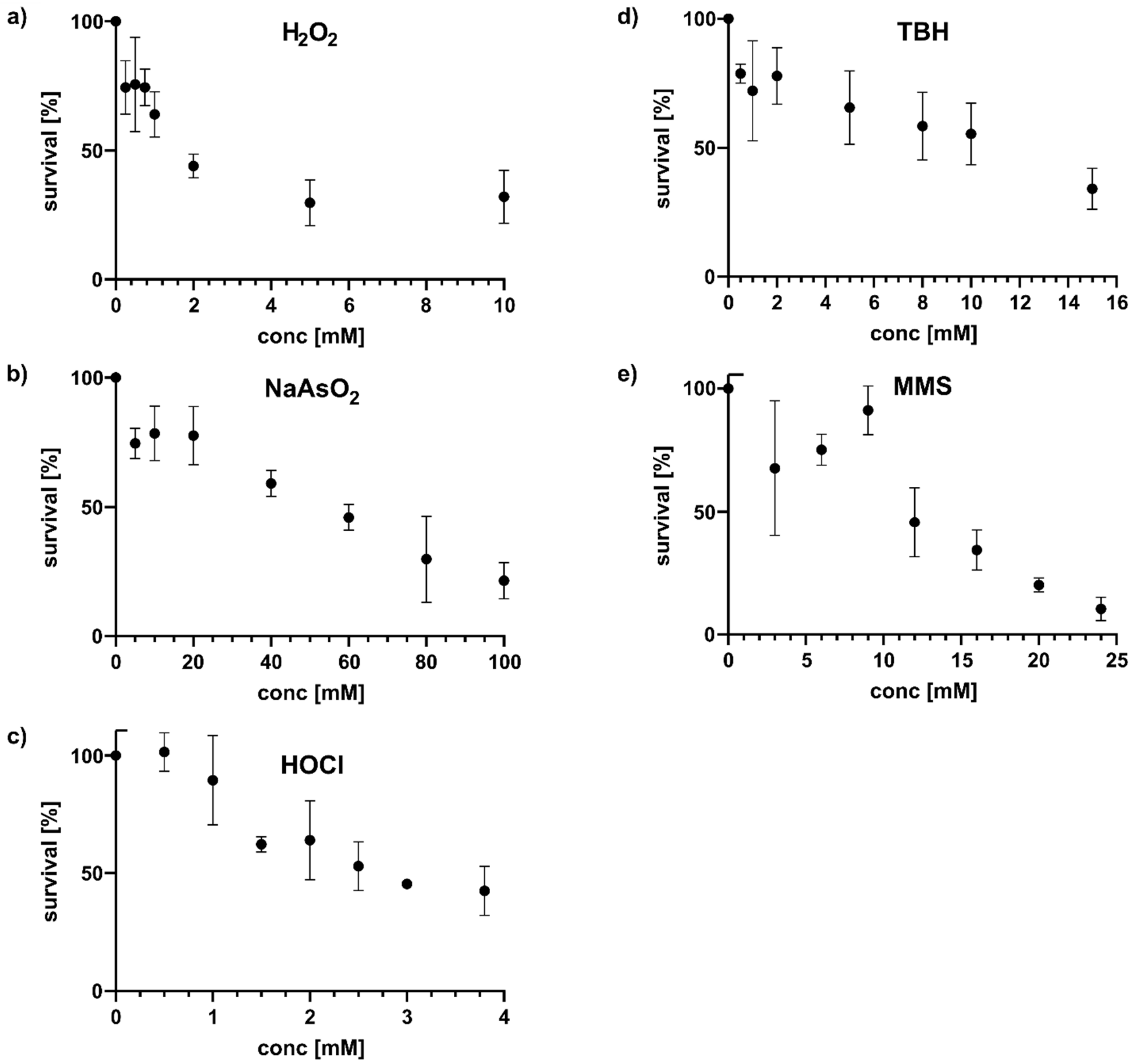

Figure S1: *LD_{50} for* S. cerevisiae *BY4741 after 60 min of stress exposure to chemicals with different concentrations a) H2O2: hydrogen peroxide; 0, 0.25, 0.5, 0.75, 1, 2, 5, 10 mM b) NaAsO2: sodium arsenite; 0, 5, 10, 20, 40, 60, 80, 100 mM c) HOCl: hypochloric acid; 0, 0.5, 1, 1.5, 2, 2.5, 3, 3.8 mM d) TBH: tert-butyl hydroperoxide; 0, 0.5, 1, 2, 5, 8, 10, 15 mM e) MMS: methyl-methanesulfonate; 0, 3, 6, 9, 12, 16, 20, 24 mM; determined LD_{50} concentrations: H_2O_2 (2 mM), $NaAsO_2$(40 mM), HOCl (3 mM), MMS (12 mM), TBH (10 mM); data from n=3 biological replicates with standard deviation*

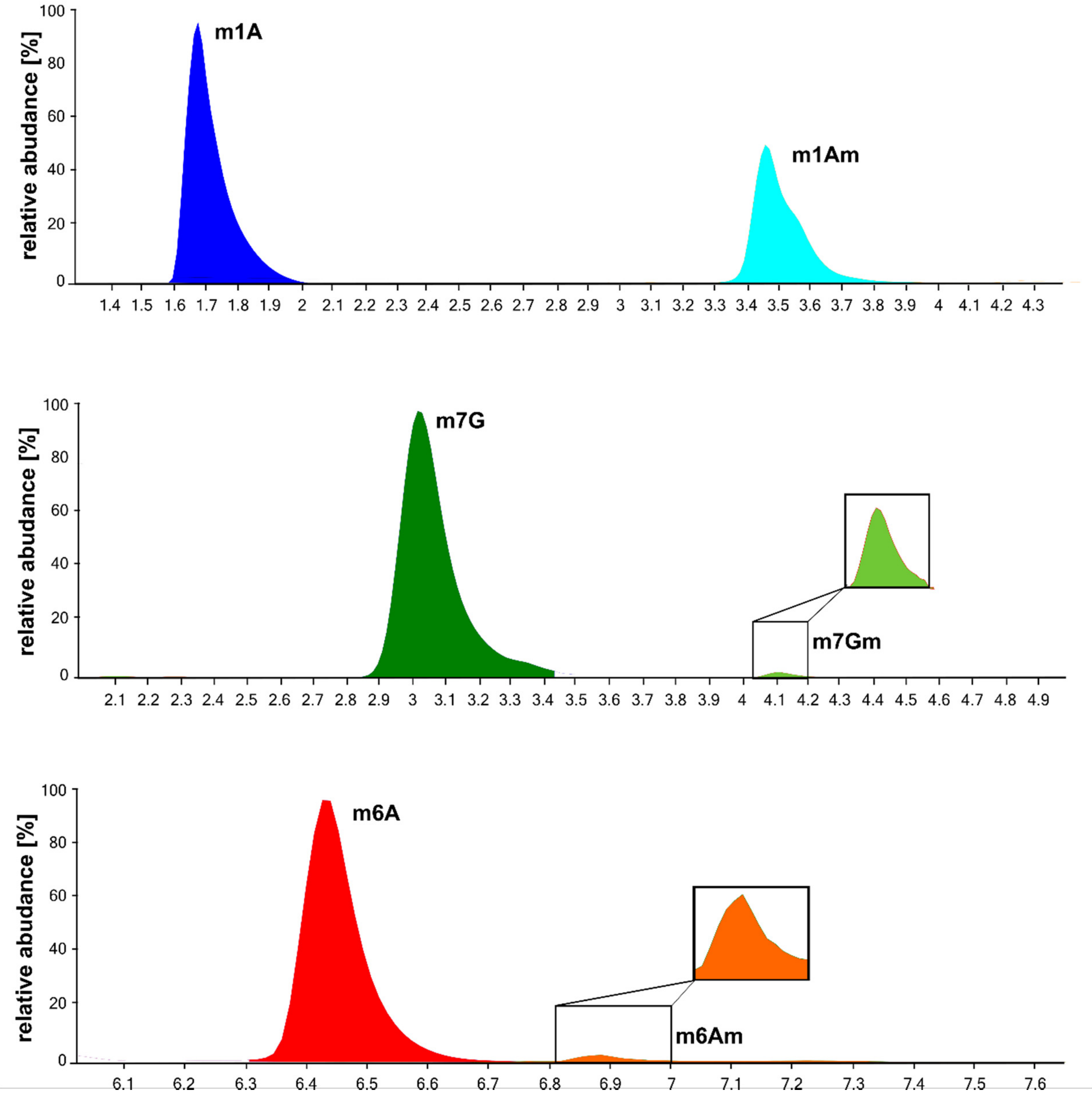

Figure S2: *Overlay of chromatograms from found damage products in total RNA after MMS treatment for 60 min. Peaks were scaled to largest in each chromatogram, m^3C (Rt: 1.65 min), m^1A (Rt: 1.67 min), m^7G (Rt: 3.02 min), m^1Am (Rt: 3.46 min), m^7Gm (Rt: 4.11 min), m^6A (Rt: 6.43), m^6Am (Rt: 6.90 min)*

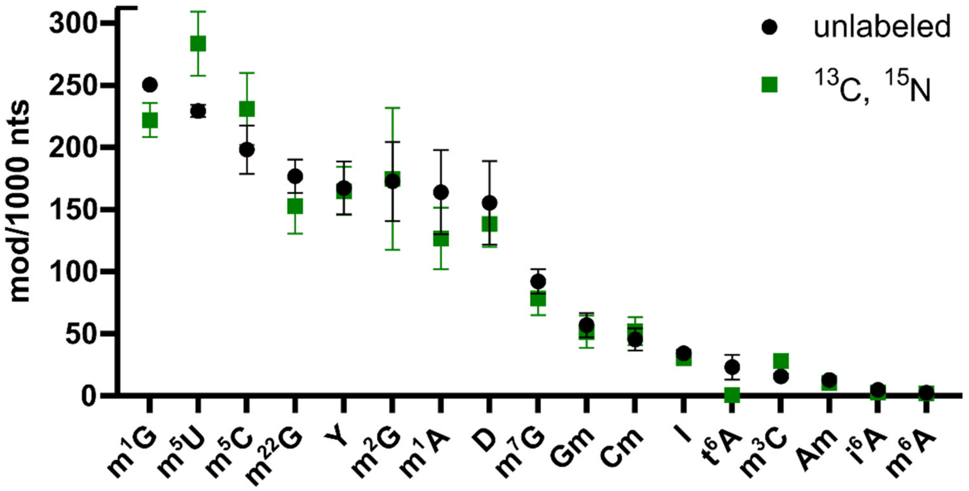

Figure S3: *comparative NAIL-MS of total RNA for labeling validation of $^{13}C_6$-glucose and $^{15}N_2$-uracil labeling (green) in comparison to unlabeled RNA (black), data from n=2 biological replicates with standard deviation*

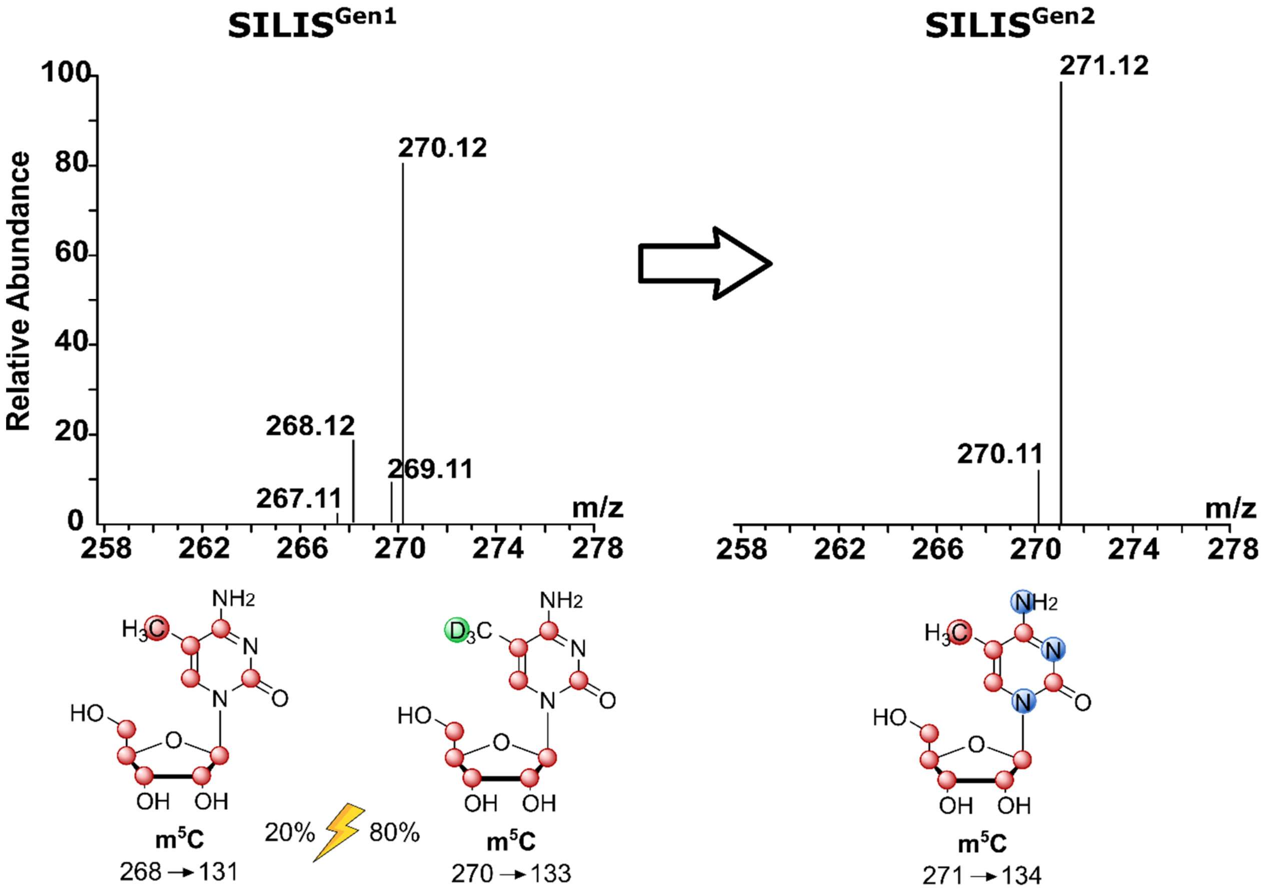

Figure S4: *Improvement of defined labeling of methylated nucleosides in SILIS^{Gen2}. Left: SILIS^{Gen1} was labeled using ^{13}C Silantes rich growth medium supplemented with ^{13}C-glucose and L-methionine-[$^{2}H_3$]-methyl which led to incomplete labeling of methylated nucleosides. Right: SILIS^{Gen2} was labeled using ^{13}C, ^{15}N Silantes rich growth medium supplemented with ^{13}C-glucose where all respective atoms are ^{13}C- and ^{15}N-labeled.*

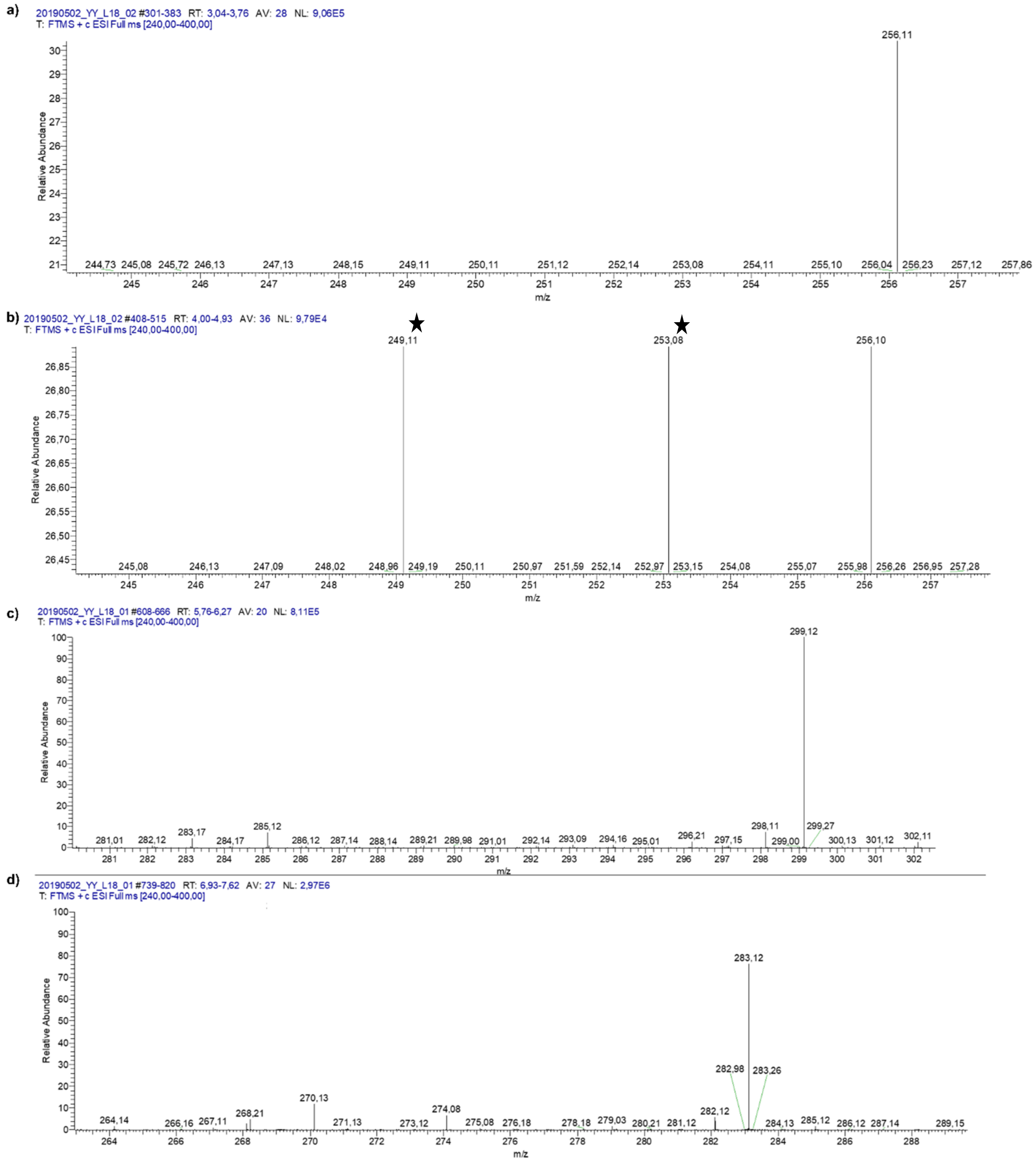

Figure S5: *High-resolution mass spectra of SILIS^{Gen2} shows representative nucleosides from the produced labeled RNA digests. a) cytosine m/z 256.11 b) uridine m/z 256.10; * indicates background contaminations c) guanosine m/z 299.12 d) adenosine m/z 283.12*

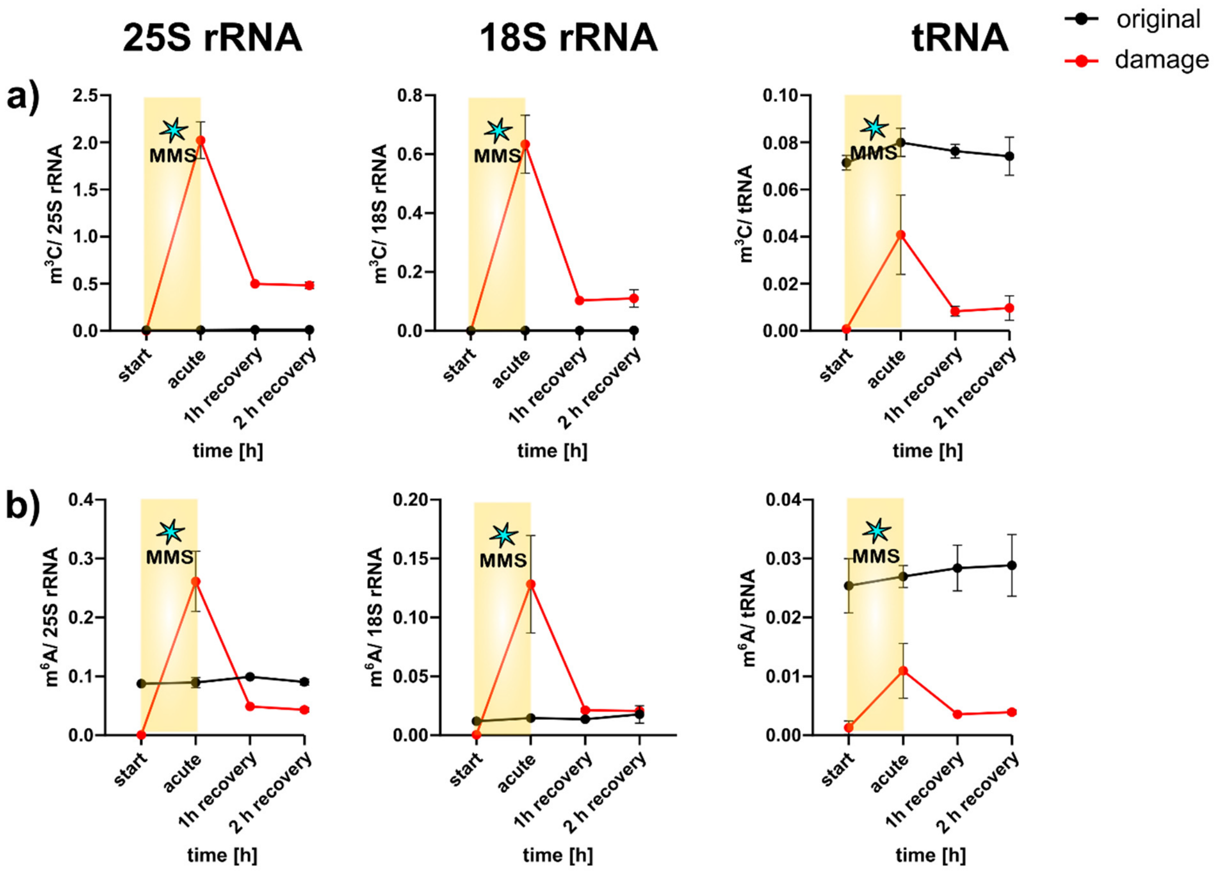

Figure S6: *Methylome discrimination based pulse chase experiment (a) number of m^3C per respective RNA (b) number of m^6A per respective RNA, red line shows damage induced methylation level, black line shows enzymatic methylation, data for tRNA from n=3 biological replicates with standard deviations, data for 25S and 18S rRNA from n=2 biological replicates with standard deviation*

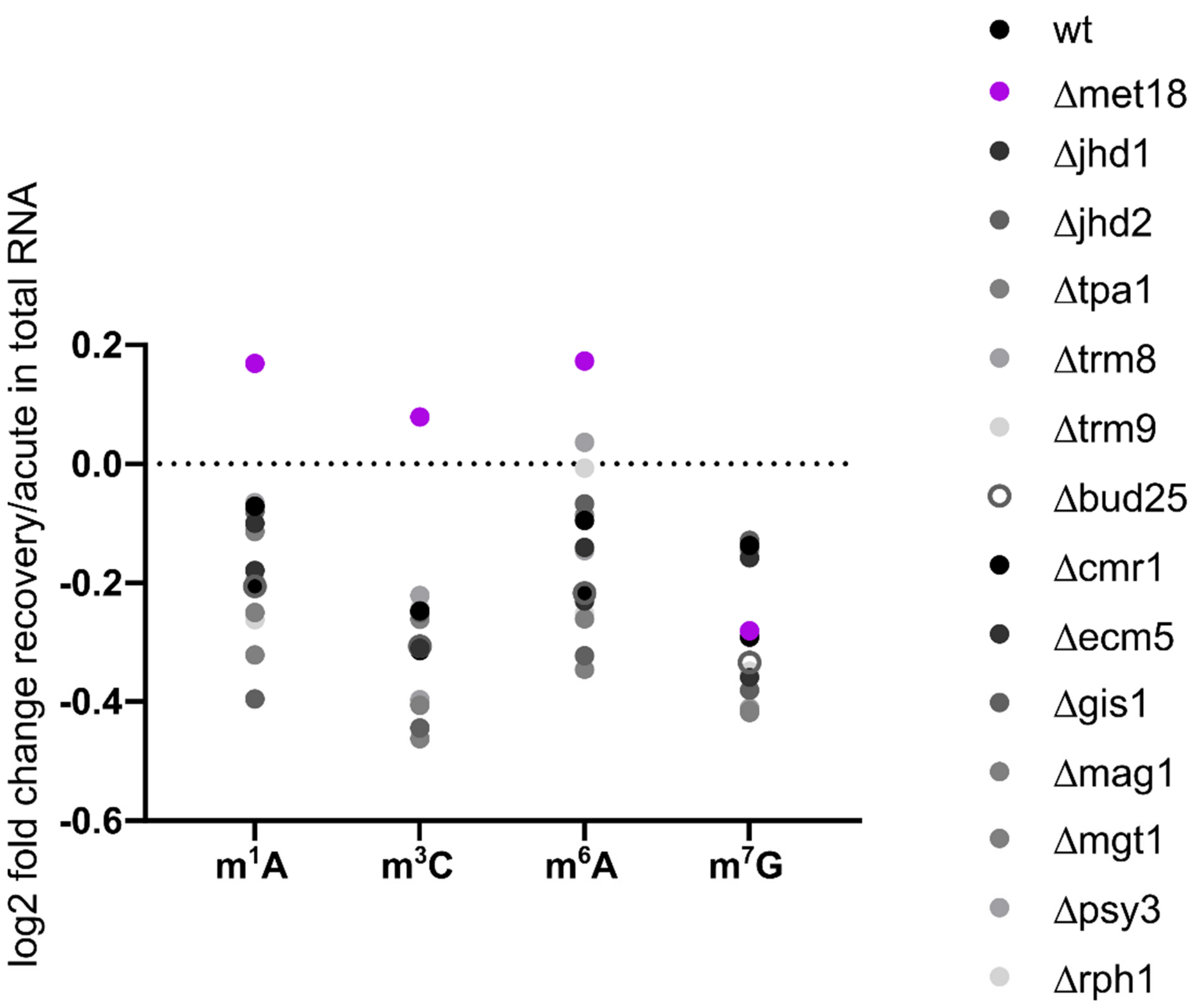

Figure S7: *log2 fold change from nucleoside abundance in acute stress phase in contrast to 1h recovery, Y < 0 indicates decrease of nucleoside level in recovery and Y > 0 indicates increase of nucleoside level in recovery, data from one biological replicate*

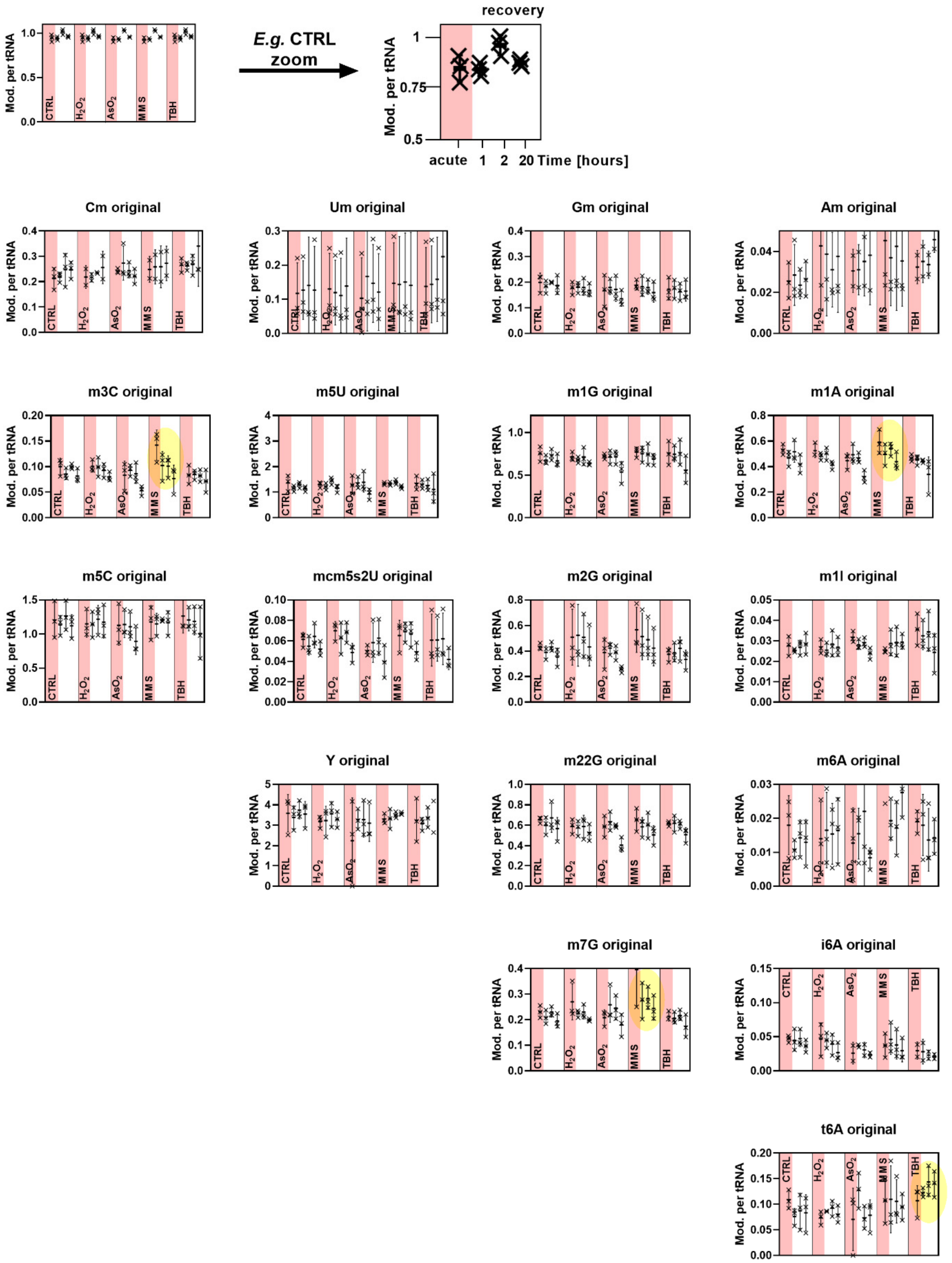

Figure S8: *Absolute abundance of modified nucleosides per original tRNA from NAIL-MS pulse chase experiment. Stress exposure phase (acute) is highlighted in red and recovery timepoints (1, 2 and 20 hrs) in white. Top plot gives an explanation for a fictive dataset. Yellow circles highlight interesting abundance changes in original tRNAs.*

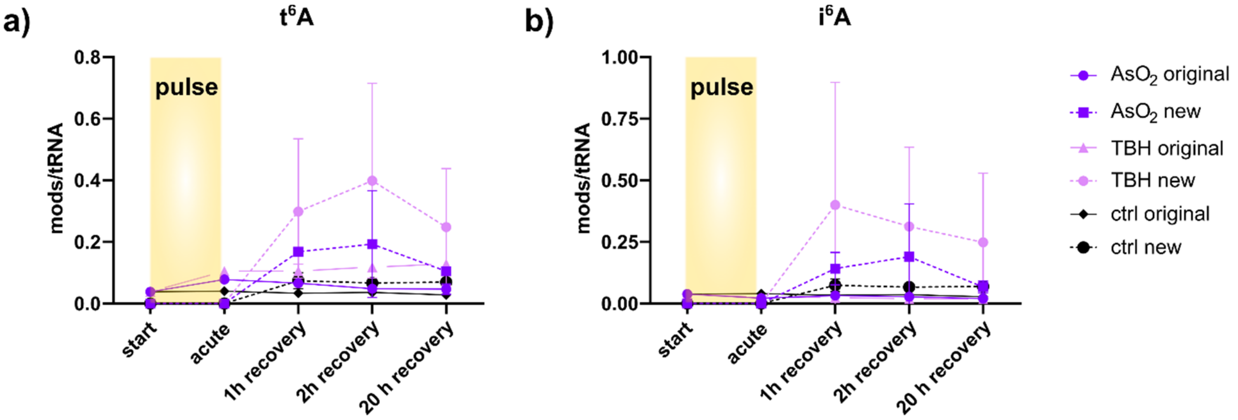

Figure S9: *NAIL-MS pulse chase experiment (a) comparison of original (solid line) and new (dashed lines) transcripts of ctrl (black), AsO_2 (violet) and TBH (pink) exposed yeast cultures. data from n=2 biological replicates with error bars reflecting standard deviation.*

Table S1: Overview of used synthetic standards, their vendors and alternative vendors. Sigma: Sigma, Aldrich, Munich, Germany; Carbosynth, Newbury, UK; TRC: Toronto Research Chemicals, Toronto, CA; Berry & Associates, Dexter, MI, USA.

Nucleoside	Abbreviation	Obtained from:	Also available from:
**cytidine**	C	Sigma	
**2′ -O-methylcytidine**	Cm	Carbosynth	
**3-methylcytidine**	m3C	Carbosynth	Carbo and TRC
**5-methylcytidine**	m5C	Carbosynth	
**2-thiocytidine**	s2C	Berry & Associates	
**N4-acetylcytidine**	ac4C	Carbosynth	
**pseudouridine**	Y	Carbosynth	
**uridine**	U	Sigma	
**dihydrouridine**	D	Apollo scientific, UK	
**2′ -O-methyluridine**	Um	Carbosynth	
**3-methyluridine**	m3U	Dedon Lab	
**5-methyluridine**	m5U	Carbosynth	
**4-thiouridine**	s4U	ordered from TRC	
**5-methoxycarbonylmethyl-2-thiouridine**	mcm5s2U	Helm Lab	
**guanosine**	G	Sigma	
**1-methylguanosine**	m1G		
**2′ -O-methylguanosine**	Gm	Carbosynth	
**N2-methylguanosine**	m2G	Dedon Lab	
**7-methylguanosine**	m7G	Carbosynth	
**N2,N2-dimethylguanosine**	m2,2G	Carbosynth	
**adenosine**	A	Sigma	
**inosine**	I	Carbosynth	
**1-methyladenosine**	m1A		Carbosynth
**2-methyladenosine**	m2A	Dedon Lab	
**2′ -O-methyladenosine**	Am	Carbosynth	Carbo and TRC
**N6-methyladenosine**	m6A	Carbosynth	Carbo, TRC and B&A
**N6,N6-dimethyladenosine**	m6,6A	Alfa Chemistry	TRC
**N6-isopentenyladenosine**	i6A	Dedon Lab	TRC
**N6-threonylcarbamoyladenosine**	t6A	TRC	TRC

Table S2: Comparison of S. cerevisiae 18S and 25S rRNA modification levels determined in this work and literature [Yang, J. et al. Mapping of complete set of ribose and base modifications of yeast rRNA by RPHPLC and mung bean nuclease assay. PloS one 11, e0168873 (2016).; Lovejoy, A. F., Riordan, D. P. & Brown, P. O. Transcriptome-wide mapping of pseudouridines: pseudouridine synthases modify specific mRNAs in S. cerevisiae. PloS one 9, e110799 (2014).] , data from n=10 biological replicates with standard deviation, n.d. = not detected.

	literature		experimental	
**Modification**	**18S**	**25S**	**18S**	**25S**
Cm	3	7	3.06 ± 0.34	6.78 ± 0.90
Um	2	8	1.90 ± 0.30	6.44 ± 1.05
Gm	5	10	4.34 ± 0.55	8.43 ± 1.25
Am	8	12	6.03 ± 1.12	8.84 ± 3.24
Ψ	13	30	12.24 ± 1.64	27.27 ± 3.32
ac^4C	2	absent	1.23 ± 0.32	0.15 ± 0.14
m^7G	1	absent	$0.77 \pm 9.85 \cdot 10^{-2}$	$4.7 \cdot 10^{-2} \pm 1.42 \cdot 10^{-2}$
$m^6{}_2A$	2	absent	$0.75 \pm 1.52 \cdot 10^{-2}$	$2.38 \cdot 10^{-2} \pm 4.84 \cdot 10^{-3}$
$m^1acp^3\Psi$	1	absent	n.d.	n.d.
m^5C	absent	2	$0.13 \pm 5.27 \cdot 10^{-2}$	1.84 ± 0.37
m^3U	absent	2	$0.11 \pm 3.50 \cdot 10^{-2}$	1.50 ± 0.32
m^1A	absent	2	$0.15 \pm 7.76 \cdot 10^{-2}$	1.90 ± 0.20
m^3C	absent	absent	$4.12 \cdot 10^{-4} \pm 4.05 \cdot 10^{-4}$	$6.10 \cdot 10^{-3} \pm 3.53 \cdot 10^{-3}$
m^6A	absent	absent	$2.04 \cdot 10^{-2} \pm 7.28 \cdot 10^{-3}$	$0.14 \pm 3.12 \cdot 10^{-2}$
m^6Am	absent	absent	$1.40 \cdot 10^{-4} \pm 3.10 \cdot 10^{-4}$	$1.50 \cdot 10^{-4} \pm 4.50 \cdot 10^{-2}$

Table S3: absolute number of enzymatically placed methylation (original, blue) and damage methylation (damage, red) per respective RNA after 60 min of MMS treatment (n=2*)*

		m^1A		m^3C		m^6A		m^7G	
**tRNA**	**original**	0.6568	0.5542	0.0865	0.0786	0.0262	0.0291	0.3682	0.3163
	**damage**	0.3027	0.1842	0.0560	0.0438	0.0146	0.0126	0.5864	0.3841
**25S rRNA**	**original**	1.9522	1.9539	0.0029	0.0039	0.0973	0.1338	0.0477	0.0438
	**damage**	1.7528	1.9876	0.5315	0.6391	0.0992	0.1471	7.7250	8.1068
**18S rRNA**	**original**	0.1755	0.1775	0.0005	0.0004	0.0163	0.0162	0.8018	0.7478
	**damage**	0.8561	0.9351	0.3085	0.3475	0.0592	0.0618	3.2262	3.2513

Table S4: *absolute number of modification per G in total RNA after 60 min of MMS exposure (acute, yellow) and after 1h of recovery (green) in different strains of* S. cerevisiae *BY4741 (n=1)*

	**m1A**		**m3C**		**m6A**		**m7G**	
	**acute**	**1h recovery**	**acute**	**1h recovery**	**acute**	**1h recovery**	**acute**	**1h recovery**
**wt**	0.01003	0.00802	0.00262	0.00149	0.00108	0.00065	0.01373	0.00845
**Δjhd1**	0.00885	0.00703	0.00264	0.00130	0.00058	0.00042	0.01184	0.00824
**Δjhd2**	0.00883	0.00735	0.00244	0.00137	0.00029	0.00050	0.01157	0.00860
**Δtpa1**	0.00905	0.00696	0.00238	0.00131	0.00959	0.00052	0.01133	0.00818
**Δtrm8**	0.00782	0.00673	0.00214	0.00128	0.00828	0.00048	0.00810	0.00580
**Δtrm9**	0.01031	0.00864	0.00266	0.00153	0.00789	0.00054	0.00053	0.15220
**Δbud25**	0.01047	0.00648	0.00245	0.00121	0.00104	0.00063	0.01235	0.00881
**Δcmr1**	0.01150	0.00731	0.00268	0.00130	0.00101	0.00061	0.01515	0.00775
**Δecm5**	0.01123	0.00728	0.00273	0.00136	0.00112	0.00066	0.01588	0.00695
**Δgis1**	0.01502	0.00636	0.00331	0.00119	0.00136	0.00065	0.01745	0.00726
**Δmag1**	0.00591	0.00330	0.00191	0.00075	0.00060	0.00033	0.01543	0.00594
**Δmet18**	0.00493	0.00740	0.00165	0.00140	0.00041	0.00062	0.01613	0.00846
**Δmgt1**	0.00622	0.00306	0.00206	0.00071	0.00058	0.00026	0.01679	0.00641
**Δpsy3**	0.00460	0.00285	0.00152	0.00061	0.00040	0.00029	0.01372	0.00532
**Δrph1**	0.00559	0.00315	0.00185	0.00073	0.00049	0.00027	0.01372	0.00532
